# Using Highly Detailed Administrative Data to Predict Pneumonia Mortality

**DOI:** 10.1371/journal.pone.0087382

**Published:** 2014-01-31

**Authors:** Michael B. Rothberg, Penelope S. Pekow, Aruna Priya, Marya D. Zilberberg, Raquel Belforti, Daniel Skiest, Tara Lagu, Thomas L. Higgins, Peter K. Lindenauer

**Affiliations:** 1 Department of Medicine, Medicine Institute, Cleveland Clinic, Cleveland, Ohio, United States of America; 2 Division of General Medicine, Baystate Medical Center, Springfield, Massachusetts, United States of America; 3 Center for Quality of Care Research, Baystate Medical Center, Springfield, Massachusetts, United States of America; 4 Department of Medicine, Tufts University School of Medicine, Boston, Massachusetts, United States of America; 5 University of Massachusetts Amherst, Amherst, Massachusetts, United States of America; 6 EviMed Research Group, LLC, Goshen, Massachusetts, United States of America; 7 Division of Infectious Diseases, Baystate Medical Center, Springfield, Massachusetts, United States of America; 8 Division of Pulmonary and Critical Care, Baystate Medical Center, Springfield, Massachusetts, United States of America; Hôpital Robert Debré, France

## Abstract

**Background:**

Mortality prediction models generally require clinical data or are derived from information coded at discharge, limiting adjustment for presenting severity of illness in observational studies using administrative data.

**Objectives:**

To develop and validate a mortality prediction model using administrative data available in the first 2 hospital days.

**Research Design:**

After dividing the dataset into derivation and validation sets, we created a hierarchical generalized linear mortality model that included patient demographics, comorbidities, medications, therapies, and diagnostic tests administered in the first 2 hospital days. We then applied the model to the validation set.

**Subjects:**

Patients aged ≥18 years admitted with pneumonia between July 2007 and June 2010 to 347 hospitals in Premier, Inc.’s Perspective database.

**Measures:**

In hospital mortality.

**Results:**

The derivation cohort included 200,870 patients and the validation cohort had 50,037. Mortality was 7.2%. In the multivariable model, 3 demographic factors, 25 comorbidities, 41 medications, 7 diagnostic tests, and 9 treatments were associated with mortality. Factors that were most strongly associated with mortality included receipt of vasopressors, non-invasive ventilation, and bicarbonate. The model had a c-statistic of 0.85 in both cohorts. In the validation cohort, deciles of predicted risk ranged from 0.3% to 34.3% with observed risk over the same deciles from 0.1% to 33.7%.

**Conclusions:**

A mortality model based on detailed administrative data available in the first 2 hospital days had good discrimination and calibration. The model compares favorably to clinically based prediction models and may be useful in observational studies when clinical data are not available.

## Introduction

Bacterial pneumonia is a leading cause of morbidity and mortality in the United States. Every year, more than 8 million patients are admitted to US hospitals with pneumonia; 8.8% of them will die. [1] Despite the common nature of this condition, there are large gaps in our knowledge regarding how best to care for pneumonia patients. Most recommendations in national treatment guidelines are not based on randomized trials, and there is a paucity of comparative effectiveness research.

Administrative databases derived from billing records are attractive candidates for health services research, as well as for use in hospital profiling initiatives, because the number of patient records is large and the acquisition cost is low. Observational studies using administrative data can be used to assess comparative effectiveness in real world settings, and findings from such studies are sometimes confirmed in randomized trials. One concern, however, is that such studies are often biased by confounding by indication, in which the choice of treatment is influenced by a patient’s severity of illness. This threat can be limited through the use of validated risk prediction instruments that are capable of adjusting for pre-treatment severity of illness, as well as comorbidities.

There exist a number of validated pneumonia mortality prediction instruments for use in clinical care. [2], [3] All of these require clinical data, such as respiratory rate or blood urea nitrogen, which are not generally available in administrative data sets. Others have attempted to construct predictive mortality models from administrative data. International Classification of Diseases, Ninth Revision, Clinical Modification (ICD-9-CM) codes assigned at discharge are highly predictive of mortality, in great part because they include complications of hospitalization which often precede death. [4] Such models are not useful for severity adjustment because they incorporate the results of treatment (e.g., complications) as predictors. Models restricted to demographics and comorbidities at the time of admission have much lower predictive accuracy [5].

Highly detailed administrative datasets include a date-stamped record for each item administered during a hospitalization; this allows for differentiation between factors present at the time of hospitalization and those arising during the stay. We used one such dataset to create and validate a mortality risk prediction model that included only tests and treatments administered in the first 2 hospital days along with patient demographics and comorbidities.

## Methods

### Setting and Patients

We identified patients discharged between July 1, 2007 and June 30, 2010 from 347 US hospitals that participated in Premier, Inc.’s Perspective, a database developed for measuring quality and healthcare utilization that has been described previously. [6]–[8] Member hospitals represent all regions of the US, and are generally reflective of US hospitals; although larger hospitals, hospitals in the South and those in urban areas are over represented. Perspective contains all data elements found in the uniform billing 04 form, such as sociodemographic information, ICD-9-CM diagnosis and procedure codes, as well as hospital and physician information. It also includes a date-stamped log of all billed items and services, including diagnostic tests, medications, and other treatments. Because the data do not contain identifiable information, the Institutional Review Board at Baystate Medical Center determined that this study did not constitute human subjects research.

We included all patients aged ≥18 years with a principal diagnosis of pneumonia, or a secondary diagnosis of pneumonia paired with a principal diagnosis of respiratory failure, ARDS, respiratory arrest, sepsis or influenza (Figure S1). Diagnoses were assessed using International Classification of Diseases, Ninth Revision, Clinical Modification (ICD-9-CM) codes. We excluded all patients transferred in from or out to other acute care facilities, because we either could not assess initial severity or could not assess outcomes; those with a length of stay of 1 day or less; patients with cystic fibrosis; those whose attending physician of record was in a specialty that would not be expected to treat pneumonia (e.g., psychiatry); those with a diagnosis related grouping (DRG) inconsistent with pneumonia (e.g., Prostate OR procedure); those with a code indicating that the pneumonia was not present on admission; and any patient who did not have a chest radiograph and did not begin antibiotics on hospital day 1 or 2. For patients with multiple eligible admissions in the study period, 1 admission was randomly selected for inclusion.

### Markers of Comorbid Illness and Pneumonia Severity

For each patient, we extracted age, gender, race/ethnicity, insurance status, principal diagnosis, comorbidities, and specialty of the attending physician. Comorbidities were identified from ICD-9-CM secondary diagnosis codes and DRGs using Healthcare Cost and Utilization Project Comorbidity Software, version 3.1, based on the work of Elixhauser. [9] We identified a group of medications, tests, and services that are typically associated with chronic medical conditions (e.g., spironolactone, warfarin, need for a special bed to reduce pressure ulcers), as well as acute medications that may indicate severe illness (e.g., vasopressors, intravenous steroids). We also identified early use of diagnostic tests (e.g., arterial blood gas, serum lactate) and therapies (e.g., mechanical ventilation, blood transfusion, restraints) that are associated with more severe presentations of pneumonia. The complete list of medications, tests, and treatments appears in Table S1. To avoid conflating initial severity with complications of treatment, we limited our analysis to those markers received in the first 2 hospital days. We used the first 2 days because hospital days are demarcated at midnight and the first day often represents only a few hours.

### Statistical Analysis

Individual predictors of mortality were assessed using Chi-square tests using the full study cohort. Stratifying by hospital, 80% of the eligible admissions were randomly assigned to a derivation and 20% to a validation cohort, and the two cohorts were compared for differences in potential predictors. Using the derivation cohort, we developed a series of multivariable logistic regression models to predict in-hospital death. Hierarchical generalized linear models (HGLM) with a logit link (SAS PROC GLIMMIX) were used to account for the clustering of patients within hospitals. We grouped predictors into the following categories: demographics, comorbid conditions, and severity markers. We developed separate mortality models for each of these categories, including main effects and significant pairwise interactions. Factors significant at p<0.05 were retained. For each model we calculated the area under the receiver operating characteristic (AUROC) curve, together with 95% confidence intervals. [10] The final model was developed by sequentially adding effects retained in individual category models and evaluating pairwise interaction terms. Main effects that were dropped at earlier stages were re-evaluated for inclusion in the final model.

The purpose of the model was accurate prediction of mortality and risk stratification. We did not attempt to determine which individual factors were associated with mortality or to imply causality. Therefore, we did not require a priori information about the association of the various risk factors or interaction terms with the outcome. Although such an approach may result in spurious associations of individual risk factors, it need not necessarily detract from the model’s accuracy of prediction, which was our primary concern [11].

In order to guard against the possibility of overfitting our model, parameter estimates derived from the model were used to compute individual mortality risk in the remaining 20% of the admissions (the validation cohort). Discrimination of the final model in the validation set was assessed by the c-statistic as well as the expected/observed ratio. Both cohorts were categorized by decile of risk based on the probability distribution in the derivation cohort, and observed mortality was compared to that predicted by the model. We also used the integrated discrimination improvement (IDI) index [12] to measure the improvement of the final model over a basic model including only demographics and ICD-9-CM comorbidities.

We next evaluated model performance in subpopulations of the entire cohort based on hospital and patient characteristics. Specifically, we assessed model performance in strata defined by hospital size, teaching status, patient age, ICU and non-ICU admissions, and pneumonia type [healthcare-associated (HCAP) vs. community-acquired (CAP)]. All analyses were performed using the Statistical Analysis System (version 9.2, SAS Institute, Inc., Cary, NC) and STATA (StataCorp. 2007. Stata Statistical Software: Release 10. College Station, TX: StataCorp LP).

## Results

The dataset included 200,870 patients in the derivation cohort and 50,037 patients in the validation cohort. Patient characteristics of the full study cohort appear in Table 1. Most patients were over age 65, 53.3% were female and 68.0% were white. The most common comorbidities were hypertension (46.5%), diabetes (23.8%), chronic pulmonary disease (48.6%), and anemia (22.2%). Patients in the validation cohort were similar (Table S2).

**Table 1:** Patient Characteristics Associated with Inpatient Mortality.

	Discharged Alive	Died	*p*
	n (%)	n (%)	
Total	232835 (92.8)	18072 (7.2)	
Demographics			
Age, y			
18–24	3344 (98.3)	57 (1.7)	<.001
25–34	7304 (98.0)	149 (2.0)	
35–44	13209 (97.1)	396 (2.9)	
45–54	27068 (96.0)	1133 (4.0)	
55–64	37040 (94.1)	2331 (5.9)	
65–74	46217 (92.8)	3587 (7.2)	
75–84	57140 (91.1)	5598 (8.9)	
85^+^	41513 (89.6)	4821 (10.4)	
Gender			
Female	124878 (93.4)	8846 (6.6)	<.001
Male	107957 (92.1)	9226 (7.9)	
Race/Ethnicity			
White	158251 (92.8)	12334 (7.2)	<.001
Black	27436 (93.7)	1853 (6.3)	
Hispanic	11417 (93.2)	829 (6.8)	
Other	35731 (92.1)	3056 (7.9)	
Marital status			
Married	88933 (93.0)	6743 (7.0)	<.001
Single	118828 (92.9)	9144 (7.1)	
Other/Missing	25074 (92.0)	2185 (8.0)	
Insurance payor			
Medicare	155340 (91.7)	14143 (8.3)	<.001
Medicaid	19457 (94.4)	1155 (5.6)	
Managed care	33300 (95.5)	1564 (4.5)	
Commercial-Indemnity	8990 (94.7)	502 (5.3)	
Other	15748 (95.7)	708 (4.3)	
Comorbidities			
Metastatic cancer	5493 (82.4)	1177 (17.6)	<.001
Weight loss	13584 (85.6)	2293 (14.4)	<.001
Acquired immune deficiency syndrome	60 (88.2)	8 (11.8)	0.15
Peptic ulcer disease without bleeding	46 (88.5)	6 (11.5)	0.23
Liver disease	4418 (89.8)	500 (10.2)	<.001
Solid tumor without metastasis	6315 (90.0)	701 (10.0)	<.001
Chronic blood loss anemia	1486 (90.2)	162 (9.8)	<.001
Pulmonary circulation disease	11155 (90.5)	1173 (9.5)	<.001
Congestive heart failure	44861 (90.7)	4618 (9.3)	<.001
Lymphoma	2869 (90.9)	288 (9.1)	<.001
Paralysis	6061 (91.8)	543 (8.2)	0.001
Peripheral vascular disease	13038 (92.0)	1132 (8.0)	<.001
Other neurological disorders	23753 (92.6)	1897 (7.4)	0.21
Valvular disease	14635 (92.7)	1154 (7.3)	0.59
Deficiency anemias	51929 (93.2)	3815 (6.8)	<.001
Chronic pulmonary disease	113818 (93.4)	8072 (6.6)	<.001
Alcohol abuse	5884 (93.7)	393 (6.3)	0.004
Hypothyroidism	27213 (94.3)	1638 (5.7)	<.001
Diabetes	56398 (94.5)	3311 (5.5)	<.001
Hypertension	110429 (94.7)	6145 (5.3)	<.001
Rheumatoid arthritis/Collagen vascular disease	7603 (94.7)	422 (5.3)	<.001
Depression	25111 (95.7)	1133 (4.3)	<.001
Psychoses	9670 (95.9)	409 (4.1)	<.001
Obesity	19606 (96.3)	758 (3.7)	<.001
Drug abuse	4648 (97.5)	121 (2.5)	<.001
Chronic Kidney Disease			
ICD 585.4 (Stage IV - Severe)	3106 (87.2)	457 (12.8)	<.001
ICD 585.5 (Stage V)	551 (88.6)	71 (11.4)	<.001
ICD 585.9 (Unspecified)	20339 (88.7)	2583 (11.3)	<.001
ICD 585.3 (Stage III - Moderate)	7353 (91.3)	700 (8.7)	<.001
ICD 585.2 (Stage II - Mild)	1300 (94.0)	83 (6.0)	0.08
ICD 585.1 (Stage I)	154 (94.5)	9 (5.5)	0.41
Markers of chronic disease^a^			
Vitamin K	4378 (77.7)	1260 (22.3)	<.001
Tube feeds	2094 (79.3)	548 (20.7)	<.001
Total parenteral nutrition	2481 (80.2)	612 (19.8)	<.001
Mannitol	169 (80.5)	41 (19.5)	<.001
Packed red blood cells	12946 (81.5)	2934 (18.5)	<.001
Unfractionated heparin treatment	3618 (82.2)	783 (17.8)	<.001
Ammonia	5451 (82.4)	1165 (17.6)	<.001
Lactulose (>30 gm/day)	1917 (84.9)	340 (15.1)	<.001
Special bed	827 (85.2)	144 (14.8)	<.001
Anti-arrhythmics	10354 (85.4)	1772 (14.6)	<.001
Megace	3350 (86.7)	514 (13.3)	<.001
Zinc	1797 (86.7)	276 (13.3)	<.001
Nutritional supplements	8292 (87.1)	1233 (12.9)	<.001
Oral sodium bicarbonate	1372 (87.3)	199 (12.7)	<.001
Digoxin	18149 (88.8)	2284 (11.2)	<.001
Thiamine	5902 (89.8)	669 (10.2)	<.001
Procrit/Epoetin	4473 (90.1)	493 (9.9)	<.001
Vitamin B2	73 (90.1)	8 (9.9)	0.35
Vitamin C	7130 (91.3)	682 (8.7)	<.001
Histamine2 blockers	24045 (91.4)	2265 (8.6)	<.001
Low molecular weight heparin treatment	10836 (91.4)	1014 (8.6)	<.001
Proton pump inhibitors	122825 (91.6)	11234 (8.4)	<.001
Vitamin B - folic acid	13660 (91.9)	1212 (8.1)	<.001
Calcitriol	1320 (92.0)	115 (8.0)	0.23
Vitamin A	106 (92.2)	9 (7.8)	0.80
Vitamin B6	786 (92.5)	64 (7.5)	0.71
Ferrous sulphate (>325 mg/day)	7133 (92.9)	548 (7.1)	0.81
Multi-vitamins	32291 (93.0)	2417 (7.0)	0.06
Vitamin B combination	3105 (93.1)	230 (6.9)	0.49
Spironolactone/Eplerenone	5506 (93.3)	393 (6.7)	0.10
Inhaled steroids	9437 (93.7)	630 (6.3)	<.001
Vitamin B12	3447 (93.7)	233 (6.3)	0.040
Alzheimer medications	13343 (93.8)	888 (6.2)	<.001
Aspirin	74529 (93.9)	4815 (6.1)	<.001
Carvedilol	15273 (94.3)	927 (5.7)	<.001
Parkinson medications	7304 (94.3)	443 (5.7)	<.001
Beta blockers	52491 (94.4)	3125 (5.6)	<.001
Oral calcium	18678 (94.4)	1099 (5.6)	<.001
Theophylline/Aminophylline	4156 (94.5)	244 (5.5)	<.001
Vitamin E	1445 (94.6)	82 (5.4)	0.006
Anti-depressants	59680 (94.6)	3374 (5.4)	<.001
Warfarin	19603 (94.8)	1071 (5.2)	<.001
Gastrontestinal/Antispasmodics	1633 (94.8)	89 (5.2)	0.001
Vitamin D	12545 (94.8)	691 (5.2)	<.001
Meglitinides	901 (94.8)	49 (5.2)	0.015
Tiotropium	13470 (95.0)	716 (5.0)	<.001
Oxybutynin	1809 (95.0)	96 (5.0)	<.001
Statins	64793 (95.2)	3240 (4.8)	<.001
Calcium channel blockers	27120 (95.7)	1231 (4.3)	<.001
Clonidine	9135 (95.7)	412 (4.3)	<.001
Angiotensin-converting enzyme (ACE) inhibitors	43996 (95.9)	1864 (4.1)	<.001
Salmeterol	22837 (95.9)	974 (4.1)	<.001
Nadolol	510 (95.9)	22 (4.1)	0.006
Doxazosin	2569 (95.9)	109 (4.1)	<.001
Cod liver oil	847 (96.3)	33 (3.8)	<.001
Sulfonylureas	13777 (96.4)	519 (3.6)	<.001
Thiazolidinediones	4913 (96.4)	185 (3.6)	<.001
Muscle relaxants	8138 (96.4)	302 (3.6)	<.001
Angiotensin-II receptor blockers (ARB)	20206 (96.5)	724 (3.5)	<.001
Dipeptidyl peptidase IV inhibitors	1764 (96.8)	59 (3.2)	<.001
Thiazide diuretics	14736 (97.5)	378 (2.5)	<.001
Biguanides	10789 (97.5)	276 (2.5)	<.001
Alpha-glucosidase inhibitors	119 (97.5)	3 (2.5)	0.043
Nicotine replacement therapy	11256 (97.6)	280 (2.4)	<.001
Other infections (Present on admission)			
Other infections	3453 (84.8)	618 (15.2)	<.001
Urinary tract infection	31044 (88.6)	3984 (11.4)	<.001
Empyema/Lung abscess	2224 (89.0)	275 (11.0)	<.001
Pansinusitis/Sinusitis	3225 (96.9)	103 (3.1)	<.001
ICU variables^a^			
Intensive care unit	38192 (82.0)	8359 (18.0)	<.001
Intensive care unit (observation, CVICU)	7967 (82.0)	1747 (18.0)	<.001
Intermediate care admission (step down)	4405 (94.1)	275 (5.9)	<.001
Markers of Initial Severity^a^			
Dobutamine	1072 (69.4)	473 (30.6)	<.001
Bicarbonate	6116 (70.4)	2568 (29.6)	<.001
Vasopressors	15249 (72.0)	5928 (28.0)	<.001
Pulmonary artery catheter	178 (72.1)	69 (27.9)	<.001
IV Calcium	5425 (74.8)	1827 (25.2)	<.001
Restraints	2016 (78.0)	569 (22.0)	<.001
Not able to take oral medications	22582 (82.5)	4779 (17.5)	<.001
Benzodiazepenes	26279 (84.3)	4886 (15.7)	<.001
Foley	25858 (85.1)	4524 (14.9)	<.001
Unfractionated heparin prophylaxis	28142 (90.3)	3007 (9.7)	<.001
Anti-cholinergics/Histamines	11411 (93.2)	829 (6.8)	0.06
Anti-emetics	21441 (93.3)	1552 (6.7)	0.005
Meperidine	2225 (93.4)	156 (6.6)	0.22
Low molecular weight heparin prophylaxis	83079 (93.6)	5709 (6.4)	<.001
Acetaminophen	121316 (94.9)	6467 (5.1)	<.001
Ketorolac	11701 (97.5)	298 (2.5)	<.001
Antibiotics			
Vancomycin, linezolid, or quinupristin/dalfopristin	57871 (86.0)	9454 (14.0)	<.001
Anti-pseudomonal cephalosporin, carbapenem, beta-lactam, or aztreonam	75502 (87.2)	11124 (12.8)	<.001
Anti-pseudomonal quinolone or aminoglycosides	106741 (92.1)	9220 (7.9)	<.001
Respiratory quinolone	125492 (93.0)	9460 (7.0)	<.001
Macrolide or respiratory quinolone	200329 (93.8)	13144 (6.2)	<.001
Beta-lactam, 3^rd^-generation cephalosporin, or non-pseudomonal carbapenem	117650 (94.6)	6695 (5.4)	<.001
3^rd^-generation cephalosporin or non-pseudomonal beta-lactam	118016 (94.6)	6699 (5.4)	<.001
Macrolide or doxycycline	107480 (95.0)	5649 (5.0)	<.001
Oral steroids (in prednisone equivalent dose)			
No PO steroid	210588 (92.6)	16916 (7.4)	<.001
<10 mg	2682 (94.5)	156 (5.5)	
≥10 mg & ≤80 mg	16265 (95.2)	818 (4.8)	
>80 mg	3300 (94.8)	182 (5.2)	
IV steroids (in prednisone equivalent dose)			
No IV steroid	168258 (92.9)	12829 (7.1)	<.001
<10 mg	78 (94.0)	5 (6.0)	
≥10 mg & ≤120 mg	2983 (90.6)	309 (9.4)	
>120 mg	61516 (92.6)	4929 (7.4)	
Markers of acute or chronic disease^a^			
Pentazocine	60 (87.0)	9 (13.0)	0.06
Loop diuretics	64968 (89.9)	7310 (10.1)	<.001
Opiates	54723 (90.9)	5466 (9.1)	<.001
Insulin	62312 (90.9)	6263 (9.1)	<.001
Ipratropium	114595 (92.5)	9334 (7.5)	<.001
Anti-psychotics	19728 (92.6)	1572 (7.4)	0.29
Albuterol	125306 (92.9)	9642 (7.1)	0.23
Zolpidem	20974 (96.6)	747 (3.4)	<.001
Non-steroidal anti-inflammatory drugs	18682 (96.9)	598 (3.1)	<.001
Tests and therapies^a^			
Platelets	81 (64.8)	44 (35.2)	<.001
Plasma	703 (69.5)	308 (30.5)	<.001
Arterial line	1720 (74.6)	585 (25.4)	<.001
Central line	4713 (75.1)	1561 (24.9)	<.001
Invasive mechanical ventilation	18807 (75.9)	5979 (24.1)	<.001
Non-invasive ventilation	17164 (83.4)	3409 (16.6)	<.001
Blood lactate	40150 (86.7)	6146 (13.3)	<.001
Arterial & venous blood gas	83752 (87.2)	12298 (12.8)	<.001
Pleural fluid analysis	1714 (88.0)	234 (12.0)	<.001
Head CT	30575 (88.9)	3826 (11.1)	<.001
Abdominal CT	15853 (89.7)	1813 (10.3)	<.001
Urine cultures	90452 (90.0)	10102 (10.0)	<.001
Brain natriuretic peptide	108369 (90.7)	11047 (9.3)	<.001
Sputum cultures	34434 (91.8)	3075 (8.2)	<.001
D-dimer	30434 (92.3)	2541 (7.7)	<.001
Blood cultures	209280 (92.6)	16663 (7.4)	<.001
Cerebrospinal fluid analysis	2242 (93.1)	167 (6.9)	0.61
Hospital characteristics			
Bed size			
≤200 beds	47040 (94.2)	2889 (5.8)	<.001
201–400 beds	90461 (92.5)	7357 (7.5)	
400^+^ beds	95334 (92.4)	7826 (7.6)	
Rural/Urban status			
Urban	201477 (92.7)	15950 (7.3)	<.001
Rural	31358 (93.7)	2122 (6.3)	
Teaching status			
Non-teaching	152705 (93.1)	11283 (6.9)	<.001
Teaching	80130 (92.2)	6789 (7.8)	
Region			
Northeast	37590 (91.6)	3447 (8.4)	<.001
Midwest	51779 (93.6)	3522 (6.4)	
West	39471 (92.4)	3248 (7.6)	
South	103995 (93.0)	7855 (7.0)	

a within first 2 hospital days.

### Model Development

**Figure 1:**
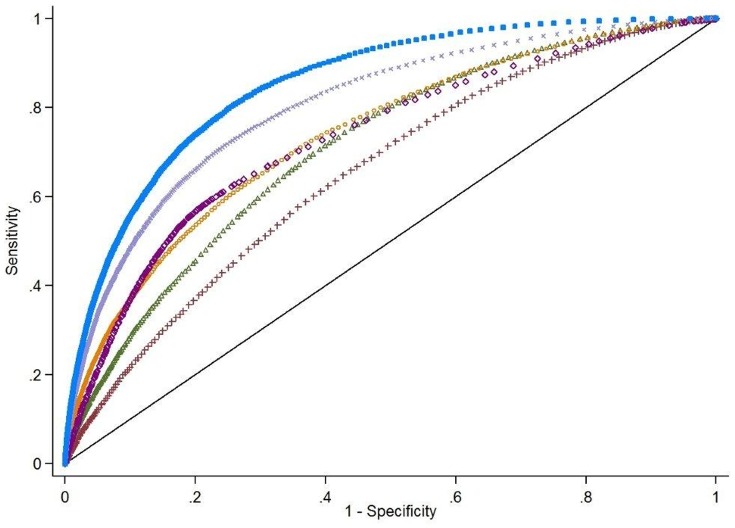
Comparison of Model Components’ Discrimination in the Derivation Cohort. Factors not significant at p<.05 and interaction terms are not included. All medications, tests and therapies are within the first 2 hospital days. Legend includes area under the ROC curve and 95% confidence intervals.

Overall in-hospital mortality in the derivation cohort was 7.2%. A large number of patient and hospital factors were associated with mortality (Table 1). Due to the large sample size, even weak associations appear highly statistically significant. Figure 1 shows the model discrimination, as measured by the area under the ROC curve, when subgroups of factors were used to model mortality. Including only patient demographics produced a model with poor discrimination (AUROC = 0.66). Using traditional ICD-9-CM based measures of comorbidity showed greater discrimination (AUROC = 0.71), as did a model that used admission to the ICU in day 1 or 2 as the only predictor (AUROC = 0.73). As an alternative measure of comorbidity, chronic medications were superior to ICD-9-CM codes in predicting mortality (AUROC 0.74 vs. 0.71, p<.001). Combining demographics, comorbidities, and markers of severity of illness on presentation (other infections present-on-admission, admission to ICU, the ability to take oral medications, and acute medications, tests and therapies used in first 2 days) offered excellent discrimination in the derivation cohort (AUROC = 0.85). We also assessed model discrimination using the IDI. Compared to the model including only demographics and ICD-9-CM comorbidities, the full model had an IDI which was 12 percentage points higher (16.6% vs. 4.6%, p<.001).

**Figure 2:**
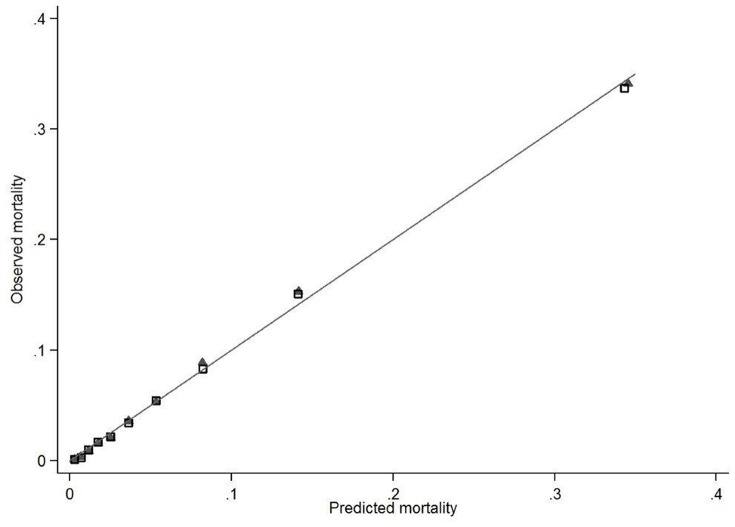
Model Calibration by Deciles of Predicted Risk in the Development and Validation Cohorts.

The final multivariable model included 3 demographic factors, 25 comorbidities, 41 medications, 7 diagnostic tests, and 9 treatments, as well as a large number of interaction terms (Table S3). The strongest predictors were early vasopressors (OR 1.71, 95% CI 1.62–1.81), early non-invasive ventilation (OR 1.55, 95% CI 1.47–1.64), and early bicarbonate treatment (OR 1.70, 95% CI 1.59–1.82). The final model produced deciles of mean predicted risk from 0.3% to 34.5%, while mean observed risk over the same deciles ranged from 0.1% to 34.1% (Figure 2).

### Validation

Model discrimination measured by the c-statistic in the validation set was 0.85 (95%CI: 0.844–0.856). Deciles of predicted risk ranged from 0.3% to 34.3% with observed risk over the same deciles from 0.1% to 33.7% (Figure 2). The expected mortality rate according to the model was 7.1% (expected/observed ratio: 1.00 [95% CI 0.97–1.03]).

Performance of the model in subpopulations of the entire cohort is shown in Table 2. The model performed well in all subpopulations tested, but discrimination was poorest among patients in intensive care (c-statistic 0.78) and best among patients aged 18 to 64 years (c-statistic 0.89). In all subgroups the range of predicted mortality extended from ≤0.3% to >90%. Model calibration was also good in all subgroups. The model tended to underestimate the risk of mortality among patients with healthcare-associated pneumonia, and to a lesser extent among patients in teaching hospitals and those outside of the ICU. At the same time, it overestimated the risk of mortality among patients with community-acquired pneumonia.

**Table 2:** Model Performance in Subpopulations of Entire Cohort.

Cohort	AUROC (95% CI)	Range of predictedmortality (%)	Expected vs. Observed (95% CI)^a^
Derivation (80%)	0.852 (0.849–0.855)	0.01–92.11	1.00 (0.98–1.02)
Validation (20%)	0.850 (0.844–0.856)	0.02–91.46	1.00 (0.97–1.03)
Hospital size			
Large Hospitals (>400 beds)	0.850 (0.846–0.854)	0.01–91.87	1.03 (1.00–1.05)
Medium (201–400 beds)	0.850 (0.846–0.854)	0.01–92.11	0.96 (0.94–0.98)
Small hospitals (≤200 beds)	0.856 (0.849–0.862)	0.01–90.56	1.00 (0.96–1.04)
Hospital teaching status			
Teaching hospitals	0.852 (0.848–0.857)	0.01–91.82	0.96 (0.94–0.98)
Non-teaching hospitals	0.851 (0.848–0.854)	0.01–92.11	1.01 (1.00–1.03)
Age groups			
Patients aged 85^+^ years	0.800 (0.793–0.806)	0.24–91.82	0.99 (0.96–1.02)
Patients aged 75–84 years	0.830 (0.824–0.835)	0.14–91.87	0.99 (0.96–1.02)
Patients aged 65–74 years	0.841 (0.834–0.845)	0.07–92.11	0.99 (0.95–1.02)
Patients aged 18–64 years	0.891 (0.887–0.896)	0.01–90.29	1.02 (0.99–1.05)
Admitted to ICU	0.775 (0.770–0.780)	0.03–92.11	1.02 (1.00–1.04)
Admitted to non-ICU care	0.829 (0.824–0.833)	0.01–91.82	0.98 (0.95–1.00)
Community acquired pneumonia	0.862 (0.859–0.866)	0.01–92.11	1.09 (1.07–1.12)
Healthcare associated pneumonia	0.814 (0.810–0.819)	0.01–91.87	0.91 (0.89–0.93)

a 95% CI: (Expected/Observed)*exp(−/+1.96*1/(√(# of deaths))).

## Discussion

In this retrospective cohort study, we used highly detailed administrative data to derive and validate a pneumonia mortality prediction model for use in observational studies. The model had discriminatory ability comparable to those derived from clinical data, but unlike most other administrative models, it included information on illness severity that would be available in the first 2 hospital days. The model also had excellent calibration and successfully divided patients into mortality deciles ranging from <0.5% to >33%. Interestingly, the 30% of patients with the lowest predicted mortality had an observed mortality of <1%.

At least two clinical prediction tools have been developed for the purposes of risk stratifying patients with community acquired pneumonia–the CURB-65, [3] modified from earlier work by the British Thoracic Society, and the Pneumonia Severity Index (PSI). [2] The CURB-65 consists entirely of exam findings and laboratory values, while the PSI incorporates some historical information as well. At least 3 studies have prospectively compared the predictive abilities of these two measures. [13]–[15] Perhaps due to differences in study population, c-statistics for predicting 30-day mortality ranged from 0.73 to 0.89 across studies; however, within any given study, there were no statistically significant differences between the two scales.

Because the clinical information required for these tools is not available in administrative databases, others have attempted to create models based solely on administrative claims. In general, such models have modest discriminatory ability, unless they are combined with laboratory data. For example, one administrative claims model developed for profiling hospitals’ pneumonia mortality rates, and containing age, sex, and 29 comorbidities (based on ICD-9-CM codes from the index hospitalization and the prior year’s outpatient visits) had a c-statistic of 0.72. [16] Addition of laboratory values to administrative data can substantially enhance discrimination. Tabak et al. demonstrated that laboratory values alone contributed 3.6 times as much explanatory power as ICD-9-CM codes and 2.5 times as much as vital signs to mortality prediction. [17] For example, the c-statistic for a model that only includes laboratory values and age was 0.80. Adding ICD-9-CM codes and vital signs increased the c-statistic to 0.82. [17] Pine et al. also found that ICD-9-CM codes alone produced a c-statistic of 0.78, whereas addition of laboratory values increased the c-statistic to 0.87. [4] Addition of chart-based data (e.g., vital signs) had a small marginal effect on the model’s predictive ability [4], [18].

Our study takes a different approach to overcoming the limitations of administrative data. In brief, our results suggest that it is possible to tell a lot about patients by the tests, medications and treatments they are prescribed. Although others have utilized ambulatory medications to predict outpatient costs and mortality, these generally do not perform better than comorbidity models based on ICD-9-CM codes. [19], [20] In contrast, by assessing medications, tests and treatments administered in the first 2 hospital days, we were able to identify chronic comorbid conditions, as well as factors indicative of the severity of illness on presentation. Indeed, use of chronic medications alone predicted mortality better than ICD-9-CM codes. This could be because billing codes are more sensitive than ICD-9-CM codes, but also because medication use can identify not just the presence of disease, but also provide information about disease severity. For example, among patients with heart failure, spironolactone often signifies severe systolic dysfunction, and nadolol in the presence of liver disease likely indicates portal hypertension. Medications, however, did not capture all the information present in ICD-9-CM codes, and the combination of the two was a more powerful predictor than either one alone. This is likely because some chronic conditions, such as metastatic cancer, may not be associated with any routine medications, but are nonetheless potent predictors of mortality.

The inclusion of certain initial tests and therapies also allowed us to estimate the severity of illness at the time of admission in the absence of laboratory or clinical data. Although it would be helpful to know the results of a blood gas, the simple presence of that test is indirect evidence that the treating physicians were concerned about a patient’s respiratory condition. Similarly, a patient receiving vasopressors is almost certainly hypotensive. More importantly, our model’s predictive ability was comparable to that seen with other administrative models that include laboratory data, as well as those that are based on physiological information obtained from review of medical records. An analogous model, designed for use in sepsis patients, demonstrated that highly detailed administrative data can achieve discrimination and calibration similar to clinical mortality prediction models, [21] with the majority of the additional explanatory power of the model arising from the inclusion of initial treatments [22].

Our study has several limitations. First, our main outcome was in-hospital mortality. Others have modeled 30-day mortality and the factors that are predictive of in-hospital mortality may be different than those which predict 30-day mortality. [23] Second, our study was conducted retrospectively and the model, therefore, may perform differently in a prospective cohort. It would certainly be premature to base treatment decisions on our model, but that is not its intended purpose. Third, our definition of pneumonia was based on diagnosis and charge codes. Some patients may not have had pneumonia and some cases of pneumonia may have been missed. These numbers are likely to be small, as the positive predictive value of an ICD-9 diagnosis paired with an antibiotic description is >95%. [24] Fourth, we excluded patients with pneumonia not present on admission, as well as transfer patients, so our model is not applicable to these groups. Finally, our model derives much of its power from physician assessments of patients’ disease, as represented by physician ordering. To the extent that prescribing thresholds vary by institution, the model may be more or less accurate in certain hospitals, and therefore could not be used for benchmarking purposes. The fact that model discrimination was good across various subgroups of hospitals is reassuring in this regard.

This model could be used in various ways. It could be used for adjustment in observational trials, including comparative effectiveness or epidemiologic studies. Although such studies might also be performed using clinical data, many institutions do not currently have the ability to automatically extract clinical data from electronic medical records and many administrative datasets do not yet contain laboratory data. Our model represents a low-cost yet accurate alternative. In addition, unlike existing clinical models, our model was validated in several different sub-populations, with excellent performance in small and large hospitals, and in teaching and non-teaching institutions.

The model could, for example, be used for severity-adjustment in a study to compare effectiveness of guideline recommended therapies to alternative treatment options in community acquired pneumonia. It could also be used to study the severity of an illness such as healthcare associated pneumonia, in which multiple comorbid illnesses might contribute to poor outcomes. It could have application for studying the methods of hospital profiling for public reporting (e.g., testing alternative definitions of diagnosis), but may not be useful for profiling hospitals per se, because thresholds for treatment might vary across hospitals making patients appear more or less sick. Finally, some aspects of the model–specifically the chronic medications–could be incorporated into clinical prediction rules such as the PSI, in order to improve their accuracy. To avoid showering clinicians with unnecessary complexity, these could be embedded in clinical information systems to provide prognostic information at the point of care. However, prospective validation of such a hybrid model is required before it can be applied in clinical care.

In conclusion, we have created a mortality prediction model based on highly detailed administrative data available in the first 2 days of hospitalization. The performance of the model was comparable to that of models based on clinical data, and the performance was consistent across different patient subpopulations. The model should be useful for comparative effectiveness research using large, administrative databases.

## Supporting Information

Figure S1 **Flow Diagram of Patient Selection.** PN – Pneumonia; ARDS – Acute Respiratory Distress Syndrome; CXR – Chest X-Ray; CH CT – Chest CT; ABX – Antibiotic; LOS – Length of Stay; MS DRG – Medicare Diagnosis Related Group; POA – Present on Admission.
(DOCX)

Table S1 **Complete List of Medications, Tests, and Treatments.**
(DOCX)

Table S2 **Patient Characteristics in the Derivation and Validation Cohorts.**
(DOCX)

Table S3 **HGLM Estimates from Multivariable Mortality Model.**
(DOCX)
