# Supplementary material for: Using Highly Detailed Administrative Data to Predict Pneumonia Mortality
Source: PLoS One. 2014 Jan 31;9(1):e87382. doi: 10.1371/journal.pone.0087382 (PMC3909106; doi:10.1371/journal.pone.0087382)
Supplement: Figure S1 — Flow Diagram of Patient Selection. PN – Pneumonia; ARDS – Acute Respiratory Distress Syndrome; CXR – Chest X-Ray; CH CT – Chest CT; ABX – Antibiotic; LOS – Length of Stay; MS DRG – Medicare Diagnosis Related Group; POA – Present on Admission. (DOCX) [file pone.0087382.s001.docx]

Figure S1: Flow Diagram of Patient Selection

PN – Pneumonia; ARDS – Acute Respiratory Distress Syndrome; CXR – Chest X-Ray; CH CT – Chest CT; ABX – Antibiotic; LOS – Length of Stay; MS DRG – Medicare Diagnosis Related Group; POA – Present on Admission
