# Supplementary material for: Using Highly Detailed Administrative Data to Predict Pneumonia Mortality
Source: PLoS One. 2014 Jan 31;9(1):e87382. doi: 10.1371/journal.pone.0087382 (PMC3909106; doi:10.1371/journal.pone.0087382)
Supplement: Table S1 — Complete List of Medications, Tests, and Treatments. (DOCX) [file pone.0087382.s002.docx]

**Table S1. Complete List of Medications, Tests, and Treatments**

| **Medications** | **Tests** | **Therapies** |
| --- | --- | --- |
| Vasopressors | Arterial & venous blood gas | Non-invasive ventilation therapy |
| Bicarbonate | Blood cultures | Invasive mechanical ventilation therapy |
| Oral meds | Sputum cultures | Plasma |
| Benzodiazepenes | CSF analysis | Platelets |
| Bumetanide | Urine cultures | Central line |
| Ethacrynic acid | Pleural fluid analysis | Arterial line |
| Furosemide | Blood lactate | Restraints |
| Metolazone | Brain natriuretic peptide | Tube feeds |
| Torsemide | Abdominal CT | Total parenteral nutrition |
| Carvedilol | D-Dimer | Packed red blood cells |
| Angiotensin-converting enzyme (ACE) inhibitors | Head CT | Foley catheter |
| Angiotensin-II receptor blockers (ARB) | Ammonia | Special bed |
| Digoxin |  | Pulmonary artery catheter |
| Spironolactone |  |  |
| Eplerenone |  |  |
| Anti-arrhythmics |  |  |
| Warfarin |  |  |
| Unfractionated heparin (treatment and prophylaxis) |  |  |
| Low molecular weight heparin (treatment and prophylaxis) |  |  |
| Morphine |  |  |
| Dobutamine |  |  |
| Amrinone |  |  |
| Milrinone |  |  |
| Albuterol |  |  |
| Tiotropium |  |  |
| Ipratropium |  |  |
| Salmeterol |  |  |
| Theophylline |  |  |
| Aminophylline |  |  |
| Beclometh |  |  |
| Mometasone |  |  |
| Budesonide |  |  |
| Nadolol |  |  |
| Lactulose |  |  |
| Procrit/Epoetin |  |  |
| Calcitriol |  |  |
| Oral Sodium bicarb |  |  |
| Flurazepam |  |  |
| Meperidine |  |  |
| Meperidine/Promethazine |  |  |
| Meprobamate |  |  |
| Pentazocine |  |  |
| Thioridazine |  |  |
| Apraclonidine |  |  |
| Clonidine |  |  |
| Propoxyphene |  |  |
| Chlorphenir |  |  |
| Dexchlor |  |  |
| Diphenhydramine |  |  |
| Hydroxyzine |  |  |
| Promethazin |  |  |
| Dicyclomine |  |  |
| Hyoscyamine |  |  |
| Donnatal |  |  |
| Nifedipine |  |  |
| Doxazosin |  |  |
| Dipyridamole/Aspirin |  |  |
| Ketorolac |  |  |
| Non steroidal anti-inflammatory drugs |  |  |
| Ticlopidine |  |  |
| Oxybutynin |  |  |
| Mineral oil |  |  |
| Trimethobenz |  |  |
| Cimetidine |  |  |
| Cyclandelate |  |  |
| Ferrous sulphate |  |  |
| Varenicline |  |  |
| Alzheimer meds |  |  |
| Parkinson meds |  |  |
| Anti-psychotics |  |  |
| Calcium (PO and IV) |  |  |
| Megace |  |  |
| Zinc |  |  |
| Multi-vitamin |  |  |
| Vitamin A |  |  |
| Vitamin AD |  |  |
| Vitamin B combination |  |  |
| Vitamin B folic acid |  |  |
| Vitamin B2 |  |  |
| Vitamin B3 |  |  |
| Vitamin B6 |  |  |
| Vitamin B12 |  |  |
| Vitamin C |  |  |
| Vitamin D |  |  |
| Vitamin E |  |  |
| Vitamin K |  |  |
| Codliver |  |  |
| Thiamine |  |  |
| Statins |  |  |
| Insulin |  |  |
| Sulfonylureas |  |  |
| Biguanides |  |  |
| Thiazolidinediones |  |  |
| Alpha-Glucosidase inhibitors |  |  |
| Meglitinides |  |  |
| Dipeptidyl peptidase IV inhibitors |  |  |
| Mannitol |  |  |
| Muscle relaxants |  |  |
| Anti-depressants |  |  |
| Steroids |  |  |
| Esomeprazole |  |  |
| Lansoprazole |  |  |
| Omeprazole |  |  |
| Pantoprazole |  |  |
| Metoclopramide |  |  |
| Famotidine |  |  |
| Ranitidine |  |  |
| Nicotine replacement therapy |  |  |
| Nutritional supplements |  |  |
| Anti-MRSA Vancomycin |  |  |
| Anti-MRSA Linezolid |  |  |
| Anti-MRSA Quinupristin/Dalfopristin |  |  |
| Anti-pseudomonal Cephalosporin |  |  |
| Anti-pseudomonal Carbapenem |  |  |
| Anti-pseudomonal Beta-lactam |  |  |
| Aztreonam |  |  |
| Anti-pseudomonal Quinolone |  |  |
| Aminoglycosides |  |  |
| Beta-lactam |  |  |
| 3rd-generation Cephalosporin |  |  |
| Non-pseudomonal Carbapenem |  |  |
| Macrolide |  |  |
| Doxycycline |  |  |
| Respiratory Quinolone |  |  |
| Non-pseudomonal Beta-lactam |  |  |
