# Supplementary material for: Using Highly Detailed Administrative Data to Predict Pneumonia Mortality
Source: PLoS One. 2014 Jan 31;9(1):e87382. doi: 10.1371/journal.pone.0087382 (PMC3909106; doi:10.1371/journal.pone.0087382)
Supplement: Table S2 — Patient Characteristics in the Derivation and Validation Cohorts. (DOCX) [file pone.0087382.s003.docx]

**Table S2. Patient Characteristics in the Derivation and Validation Cohorts**

|  | **Derivation** | **Validation** | ***p*** |
| --- | --- | --- | --- |
|  | **n (%)** | **n (%)** |  |
| Total | 200870 (80.0) | 50037 (20.0) |  |
| Age, y |  |  | .20 |
| 18 - 24 | 2728 (1.4) | 673 (1.3) |  |
| 25 - 34 | 5935 (3) | 1518 (3) |  |
| 35 - 44 | 10919 (5.4) | 2686 (5.4) |  |
| 45 - 54 | 22435 (11.2) | 5766 (11.5) |  |
| 55 - 64 | 31642 (15.8) | 7729 (15.4) |  |
| 65 - 74 | 39969 (19.9) | 9835 (19.7) |  |
| 75 - 84 | 50157 (25) | 12581 (25.1) |  |
| 85+ | 37085 (18.5) | 9249 (18.5) |  |
| Gender |  |  | .46 |
| Female | 106982 (53.3) | 26742 (53.4) |  |
| Male | 93888 (46.7) | 23295 (46.6) |  |
| Race/Ethnicity |  |  | .75 |
| White | 136630 (68) | 33955 (67.9) |  |
| Black | 23440 (11.7) | 5849 (11.7) |  |
| Hispanic | 9759 (4.9) | 2487 (5) |  |
| Other | 31041 (15.5) | 7746 (15.5) |  |
| Marital status |  |  | .37 |
| Married | 76700 (38.2) | 18976 (37.9) |  |
| Single | 102311 (50.9) | 25661 (51.3) |  |
| Other/Missing | 21859 (10.9) | 5400 (10.8) |  |
| Insurance payor |  |  | .61 |
| Medicare | 135661 (67.5) | 33822 (67.6) |  |
| Medicaid | 16490 (8.2) | 4122 (8.2) |  |
| Managed care | 27886 (13.9) | 6978 (13.9) |  |
| Commercial-Indemnity | 7581 (3.8) | 1911 (3.8) |  |
| Other | 13252 (6.6) | 3204 (6.4) |  |
| Attending physician specialty |  |  | .39 |
| Critical care medicine | 1334 (0.7) | 332 (0.7) |  |
| Family practice | 34034 (16.9) | 8506 (17) |  |
| Geriatric medicine (Family practice and internal medicine) | 2436 (1.2) | 586 (1.2) |  |
| Hospital medicine | 37103 (18.5) | 9394 (18.8) |  |
| Internal medicine | 97558 (48.6) | 24281 (48.5) |  |
| Pulmonary diseases | 10978 (5.5) | 2626 (5.3) |  |
| Other specialty | 17427 (8.7) | 4312 (8.6) |  |
| Comorbidities |  |  |  |
| Congestive heart failure | 39655 (19.7) | 9824 (19.6) | .59 |
| Valvular disease | 12644 (6.3) | 3145 (6.3) | .94 |
| Pulmonary circulation disease | 9842 (4.9) | 2486 (5) | .53 |
| Peripheral vascular disease | 11356 (5.7) | 2814 (5.6) | .80 |
| Hypertension | 93161 (46.4) | 23413 (46.8) | .10 |
| Paralysis | 5310 (2.6) | 1294 (2.6) | .47 |
| Other neurological disorders | 20600 (10.3) | 5050 (10.1) | .28 |
| Chronic pulmonary disease | 97603 (48.6) | 24287 (48.5) | .83 |
| Diabetes | 47814 (23.8) | 11895 (23.8) | .88 |
| Hypothyroidism | 23074 (11.5) | 5777 (11.5) | .71 |
| Liver disease | 3966 (2) | 952 (1.9) | .30 |
| Lymphoma | 2506 (1.2) | 651 (1.3) | .34 |
| Metastatic cancer | 5365 (2.7) | 1305 (2.6) | .43 |
| Solid tumor without metastasis | 5625 (2.8) | 1391 (2.8) | .80 |
| Rheumatoid arthritis/collagen vascular disease | 6426 (3.2) | 1599 (3.2) | .97 |
| Obesity | 16360 (8.1) | 4004 (8) | .30 |
| Weight loss | 12699 (6.3) | 3178 (6.4) | .81 |
| Chronic blood loss anemia | 1335 (0.7) | 313 (0.6) | .33 |
| Deficiency anemias | 44647 (22.2) | 11097 (22.2) | .81 |
| Alcohol abuse | 5041 (2.5) | 1236 (2.5) | .61 |
| Drug abuse | 3830 (1.9) | 939 (1.9) | .66 |
| Psychoses | 8144 (4.1) | 1935 (3.9) | .06 |
| Depression | 21098 (10.5) | 5146 (10.3) | .15 |
| Chronic Kidney Disease |  |  |  |
| ICD 585.1 (Stage I) | 134 (0.1) | 29 (0.1) | .49 |
| ICD 585.2 (Stage II - Mild) | 1101 (0.5) | 282 (0.6) | .68 |
| ICD 585.3 (Stage III - Moderate) | 6460 (3.2) | 1593 (3.2) | .71 |
| ICD 585.4 (Stage IV - Severe) | 2873 (1.4) | 690 (1.4) | .39 |
| ICD 585.5 (Stage V) | 495 (0.2) | 127 (0.3) | .77 |
| ICD 585.9 (Unspecified) | 18305 (9.1) | 4617 (9.2) | .43 |
| Markers of chronic disease^a^ |  |  |  |
| Beta blockers | 44616 (22.2) | 11000 (22) | .27 |
| Thiazides | 12063 (6) | 3051 (6.1) | .44 |
| Calcium channel blockers | 22631 (11.3) | 5720 (11.4) | .30 |
| Aspirin | 63459 (31.6) | 15885 (31.7) | .51 |
| Angiotensin-converting enzyme (ACE) inhibitors | 36649 (18.2) | 9211 (18.4) | .40 |
| Proton Pump Inhibitors | 107506 (53.5) | 26553 (53.1) | .07 |
| Histamine2 blockers | 21126 (10.5) | 5184 (10.4) | .31 |
| Digoxin | 16381 (8.2) | 4052 (8.1) | .68 |
| Carvedilol | 12947 (6.4) | 3253 (6.5) | .65 |
| Angiotensin-II receptor blockers (ARB) | 16713 (8.3) | 4217 (8.4) | .44 |
| Spironolactone/Eplerenone | 4725 (2.4) | 1174 (2.3) | .94 |
| Antiarrhythmics | 9710 (4.8) | 2416 (4.8) | .96 |
| Warfarin | 16616 (8.3) | 4058 (8.1) | .24 |
| Unfractionated heparin treatment | 3526 (1.8) | 875 (1.7) | .92 |
| Low molecular weight heparin treatment | 9451 (4.7) | 2399 (4.8) | .40 |
| Tiotropium | 11342 (5.6) | 2844 (5.7) | .75 |
| Salmeterol | 19076 (9.5) | 4735 (9.5) | .82 |
| Theophylline/Aminophylline | 3535 (1.8) | 865 (1.7) | .64 |
| Inhaled steroids | 8079 (4) | 1988 (4) | .62 |
| Lactulose (>30gm/day) | 1836 (0.9) | 421 (0.8) | .12 |
| Nadolol | 417 (0.2) | 115 (0.2) | .33 |
| Vitamin K | 4522 (2.3) | 1116 (2.2) | .78 |
| Ammonia | 5298 (2.6) | 1318 (2.6) | .97 |
| Procrit/Epoetin | 4009 (2) | 957 (1.9) | .23 |
| Calcitriol | 1131 (0.6) | 304 (0.6) | .24 |
| Oral sodium bicarbonate | 1270 (0.6) | 301 (0.6) | .44 |
| Gastrointestinal/Antispasmodic | 1375 (0.7) | 347 (0.7) | .83 |
| Clonidine | 7589 (3.8) | 1958 (3.9) | .16 |
| Doxazosin | 2174 (1.1) | 504 (1) | .14 |
| Oxybutynin | 1525 (0.8) | 380 (0.8) | 1.00 |
| Ferrous sulphate (>325 mg/day) | 6204 (3.1) | 1477 (3) | .11 |
| Nicotine replacement therapy | 9222 (4.6) | 2314 (4.6) | .75 |
| Tube feeds | 2120 (1.1) | 522 (1) | .81 |
| Alzheimer meds | 11363 (5.7) | 2868 (5.7) | .52 |
| Parkinson meds | 6183 (3.1) | 1564 (3.1) | .58 |
| Total parenteral nutrition | 2432 (1.2) | 661 (1.3) | .045 |
| Nutritional supplements | 7630 (3.8) | 1895 (3.8) | .91 |
| Oral Calcium | 15910 (7.9) | 3867 (7.7) | .15 |
| Megace | 3086 (1.5) | 778 (1.6) | .76 |
| Special bed | 788 (0.4) | 183 (0.4) | .39 |
| Packed red blood cells | 12781 (6.4) | 3099 (6.2) | .16 |
| Zinc | 1688 (0.8) | 385 (0.8) | .12 |
| Multi-vitamin | 27899 (13.9) | 6809 (13.6) | .10 |
| Vitamin A | 104 (0.1) | 11 (0) | .005 |
| Vitamin B combination | 2681 (1.3) | 654 (1.3) | .63 |
| Vitamin B - folic acid | 11952 (6) | 2920 (5.8) | .33 |
| Vitamin B2 | 70 (0) | 11 (0) | .15 |
| Vitamin B6 | 662 (0.3) | 188 (0.4) | .11 |
| Vitamin B12 | 2945 (1.5) | 735 (1.5) | .96 |
| Vitamin C | 6310 (3.1) | 1502 (3) | .11 |
| Vitamin D | 10631 (5.3) | 2605 (5.2) | .44 |
| Vitamin E | 1240 (0.6) | 287 (0.6) | .26 |
| Cod liver oil | 707 (0.4) | 173 (0.3) | .83 |
| Thiamine | 5244 (2.6) | 1327 (2.7) | .60 |
| Statins | 54373 (27.1) | 13660 (27.3) | .30 |
| Sulfonylureas | 11398 (5.7) | 2898 (5.8) | .31 |
| Biguanides | 8864 (4.4) | 2201 (4.4) | .89 |
| Thiazolidinediones | 4115 (2) | 983 (2) | .23 |
| Alpha-glucosidase inhibitors | 108 (0.1) | 14 (0) | .019 |
| Meglitinides | 770 (0.4) | 180 (0.4) | .44 |
| Dipeptidyl peptidase IV inhibitors | 1430 (0.7) | 393 (0.8) | .08 |
| Mannitol | 174 (0.1) | 36 (0.1) | .31 |
| Muscle relaxants | 6788 (3.4) | 1652 (3.3) | .39 |
| Anti-depressants | 50660 (25.2) | 12394 (24.8) | .038 |
| Primary diagnosis |  |  |  |
| Pneumonia/Influenza | 142717 (71) | 35576 (71.1) | .51 |
| Sepsis | 40818 (20.3) | 10080 (20.1) |  |
| Respiratory failure/arrest | 17335 (8.6) | 4381 (8.8) |  |
| Other infections (Present on admission) |  |  |  |
| Urinary tract infection | 28080 (14) | 6948 (13.9) | .59 |
| Pansinusitis/Sinusitis | 2691 (1.3) | 637 (1.3) | .24 |
| Empyema/Lung abscess | 2014 (1) | 485 (1) | .50 |
| Other infections | 3270 (1.6) | 801 (1.6) | .67 |
| ICU variables^a^ |  |  |  |
| Intensive care unit | 37298 (18.6) | 9253 (18.5) | .70 |
| Intensive care unit (observation, CVICU) | 7761 (3.9) | 1953 (3.9) | .68 |
| Intermediate care admission (step down) | 3761 (1.9) | 919 (1.8) | .60 |
|  |  |  |  |
| Markers of initial severity^a^ |  |  |  |
| Oral medications |  |  | .46 |
| Yes | 179012 (89.1) | 44534 (89.0) |  |
| No | 21858 (10.9) | 5503 (11.0) |  |
| Anti-emetics | 18422 (9.2) | 4571 (9.1) | .80 |
| Acetaminophen | 102354 (51) | 25429 (50.8) | .59 |
| Unfractionated heparin prophylaxis | 25039 (12.5) | 6110 (12.2) | .12 |
| Low molecular weight heparin prophylaxis | 71084 (35.4) | 17704 (35.4) | .98 |
| Dobutamine | 1234 (0.6) | 311 (0.6) | .85 |
| Meperidine | 1941 (1) | 440 (0.9) | .07 |
| Anti-cholinergics/Histamines | 9781 (4.9) | 2459 (4.9) | .68 |
| Ketorolac | 9573 (4.8) | 2426 (4.8) | .44 |
| Restraints | 2054 (1) | 531 (1.1) | .44 |
| IV Calcium | 5787 (2.9) | 1465 (2.9) | .58 |
| Foley catheter | 24351 (12.1) | 6031 (12.1) | .67 |
| Oral steroids (in prednisone equivalent dose) |  |  |  |
| No PO steroid | 182075 (90.6) | 45429 (90.8) | .78 |
| <10 mg | 2280 (1.1) | 558 (1.1) |  |
| ≥10mg & ≤80 mg | 13714 (6.8) | 3369 (6.7) |  |
| >80 mg | 2801 (1.4) | 681 (1.4) |  |
| IV steroids (in prednisone equivalent dose) |  |  |  |
| No IV steroid | 145034 (72.2) | 36053 (72.1) | .90 |
| <10 mg | 67 (0) | 16 (0) |  |
| ≥10mg & ≤120 mg | 2625 (1.3) | 667 (1.3) |  |
| >120 mg | 53144 (26.5) | 13301 (26.6) |  |
| Pulmonary artery catheter | 207 (0.1) | 40 (0.1) | .14 |
| Bicarbonate | 6936 (3.5) | 1748 (3.5) | .66 |
| Vasopressors | 16935 (8.4) | 4242 (8.5) | .74 |
| Benzodiazepenes | 25024 (12.5) | 6141 (12.3) | .26 |
| Arterial & venous blood gas | 76839 (38.3) | 19211 (38.4) | .56 |
| Blood cultures | 180884 (90.1) | 45059 (90.1) | .99 |
| Sputum cultures | 30087 (15) | 7422 (14.8) | .41 |
| Cerebrospinal fluid analysis | 1950 (1) | 459 (0.9) | .27 |
| Urine cultures | 80579 (40.1) | 19975 (39.9) | .43 |
| Pleural fluid analysis | 1562 (0.8) | 386 (0.8) | .89 |
| Blood lactate | 37130 (18.5) | 9166 (18.3) | .39 |
| Brain natriuretic peptide | 95567 (47.6) | 23849 (47.7) | .73 |
| Abdominal CT | 14183 (7.1) | 3483 (7) | .43 |
| D-dimer | 26263 (13.1) | 6712 (13.4) | .04 |
| Head CT | 27556 (13.7) | 6845 (13.7) | .82 |
| Non-invasive ventilation | 16447 (8.2) | 4126 (8.2) | .67 |
| Invasive mechanical ventilation | 19875 (9.9) | 4911 (9.8) | .59 |
| Plasma | 804 (0.4) | 207 (0.4) | .67 |
| Platelets | 99 (0) | 26 (0.1) | .81 |
| Central line | 4998 (2.5) | 1276 (2.6) | .43 |
| Arterial line | 1864 (0.9) | 441 (0.9) | .33 |
| Antibiotics |  |  |  |
| Vancomycin, linezolid, or quinupristin/dalfopristin | 53965 (26.9) | 13360 (26.7) | .46 |
| Anti-pseudomonal cephalosporin, carbapenem, beta-lactam, or aztreonam | 69394 (34.6) | 17232 (34.4) | .65 |
| Anti-pseudomonal quinolone or aminoglycosides | 92812 (46.2) | 23149 (46.3) | .81 |
| Beta-lactam, 3^rd^-generation cephalosporin, or non-pseudomonal carbapenem | 99544 (49.6) | 24801 (49.6) | .97 |
| Macrolide or doxycycline | 90567 (45.1) | 22562 (45.1) | .99 |
| Respiratory quinolone | 107937 (53.7) | 27015 (54.0) | .31 |
| 3^rd^-generation cephalosporin or non-pseudomonal beta-lactam | 99852 (49.7) | 24863 (49.7) | .93 |
| Macrolide or respiratory quinolone | 170776 (85.0) | 42697 (85.3) | .08 |
| Markers of acute or chronic disease^a^ |  |  |  |
| Opiates | 48271 (24) | 11918 (23.8) | .32 |
| Zolpidem | 17314 (8.6) | 4407 (8.8) | .18 |
| Loop diuretics | 57764 (28.8) | 14514 (29) | .27 |
| Albuterol | 107992 (53.8) | 26956 (53.9) | .66 |
| Ipratropium | 99235 (49.4) | 24694 (49.4) | .84 |
| Non-steroidal anti-inflammatory drugs | 15477 (7.7) | 3803 (7.6) | .43 |
| Pentazocine | 61 (0) | 8 (0) | .08 |
| Anti-psychotics | 17130 (8.5) | 4170 (8.3) | .16 |
| Insulin | 54946 (27.4) | 13629 (27.2) | .60 |
| Hospital characteristics |  |  |  |
| Bed size |  |  |  |
| ≤200 beds | 39996 (19.9) | 9933 (19.9) | .95 |
| 201-400 beds | 78306 (39) | 19512 (39) |  |
| 400+ beds | 82568 (41.1) | 20592 (41.2) |  |
| Rural/Urban status |  |  |  |
| Urban | 174059 (86.7) | 43368 (86.7) | .91 |
| Rural | 26811 (13.3) | 6669 (13.3) |  |
| Teaching status |  |  |  |
| Non-teaching | 131294 (65.4) | 32694 (65.3) | .92 |
| Teaching | 69576 (34.6) | 17343 (34.7) |  |
| Region |  |  |  |
| Northeast | 32855 (16.4) | 8182 (16.4) | 1.00 |
| Midwest | 44273 (22) | 11028 (22) |  |
| West | 34205 (17) | 8514 (17) |  |
| South | 89537 (44.6) | 22313 (44.6) |  |
| Outcome |  |  |  |
| Inpatient mortality | 14516 (7.2) | 3556 (7.1) | .35 |

^a^within first 48 hours after admission
