# Supplementary material for: Using Highly Detailed Administrative Data to Predict Pneumonia Mortality
Source: PLoS One. 2014 Jan 31;9(1):e87382. doi: 10.1371/journal.pone.0087382 (PMC3909106; doi:10.1371/journal.pone.0087382)
Supplement: Table S3 — HGLM Estimates from Multivariable Mortality Model. (DOCX) [file pone.0087382.s004.docx]

**Table S3. HGLM Estimates from Multivariable Mortality Model**

| **Effect** | **Estimate** | **Standard Error** |
| --- | --- | --- |
| Intercept | -2.95 | .07 |
| Age groups |  |  |
| 18-24 years | -2.44 | 0.33 |
| 25-34 years | -2.20 | 0.22 |
| 35-44 years | -1.93 | 0.15 |
| 45-54 years | -1.45 | 0.10 |
| 55-64 years | -0.99 | 0.08 |
| 65-74 years | -0.67 | 0.07 |
| 75-84 years | -0.28 | 0.06 |
| 85+ years | Referent |  |
| Gender |  |  |
| Female | -0.16 | 0.03 |
| Male | Referent |  |
| Race/Ethnicity |  |  |
| Other | -0.06 | 0.04 |
| Black | -0.11 | 0.04 |
| Hispanic | -0.17 | 0.06 |
| White | Referent |  |
| Comorbidities |  |  |
| Congestive heart failure | 0.52 | 0.05 |
| Valvular disease | -0.13 | 0.04 |
| Pulmonary circulation disease | 0.20 | 0.09 |
| Peripheral vascular disease | 0.13 | 0.04 |
| Hypertension | -0.68 | 0.05 |
| Paralysis | 0.23 | 0.08 |
| Other neurological disorders | -0.0001 | 0.06 |
| Chronic pulmonary disease | -0.15 | 0.04 |
| Diabetes | -0.15 | 0.05 |
| Hypothyroidism | -0.29 | 0.05 |
| Liver disease | 0.21 | 0.10 |
| Lymphoma | 0.25 | 0.08 |
| Metastatic cancer | 0.57 | 0.15 |
| Solid tumor without metastasis | 0.57 | 0.09 |
| Rheumatoid arthritis/Collagen vascular disease | -0.23 | 0.10 |
| Obesity | -0.37 | 0.06 |
| Weight loss | 0.67 | 0.05 |
| Deficiency anemias | -0.31 | 0.04 |
| Drug abuse | -0.71 | 0.14 |
| Psychoses | -0.25 | 0.06 |
| Depression | -0.35 | 0.05 |
| Chronic kidney disease |  |  |
| ICD 585.3 (Stage III - Moderate) | 0.21 | 0.09 |
| ICD 585.4 (Stage IV - Severe) | 0.30 | 0.06 |
| ICD 585.5 (Stage V) | 0.03 | 0.17 |
| ICD 585.9 (Unspecified) | 0.11 | 0.03 |
| Markers of chronic disease^a^ |  |  |
| Beta blockers | -0.08 | 0.03 |
| Thiazides | -0.14 | 0.06 |
| Angiotensin-converting enzyme (ACE) inhibitors | -0.18 | 0.03 |
| Digoxin | 0.14 | 0.03 |
| Carvedilol | -0.14 | 0.05 |
| Angiotensin-II receptor blockers (ARB) | -0.22 | 0.05 |
| Anti-arrhythmics | 0.37 | 0.04 |
| Warfarin | -0.25 | 0.04 |
| Unfractionated heparin treatment | 0.23 | 0.05 |
| Salmeterol | -0.18 | 0.04 |
| Lactulose (>30gm/day) | 0.25 | 0.08 |
| Vitamin K | 0.34 | 0.04 |
| Ammonia | 0.21 | 0.05 |
| Doxazosin | -1.11 | 0.37 |
| Ferrous sulphate (>325 mg/day) | 0.02 | 0.06 |
| Nicotine replacement therapy | -0.49 | 0.07 |
| Tube feeds | 0.13 | 0.06 |
| Alzheimer meds | -0.20 | 0.04 |
| Parkinson meds | -0.24 | 0.06 |
| Total parenteral nutrition | 0.21 | 0.06 |
| Nutritional supplements | 0.12 | 0.04 |
| Megace | 0.41 | 0.06 |
| Special bed | 0.33 | 0.11 |
| Packed red blood cells | 0.33 | 0.03 |
| Vitamin B - folic acid | 0.10 | 0.04 |
| Statins | -0.24 | 0.03 |
| Anti-depressants | -0.07 | 0.03 |
| Other infections present on admission |  |  |
| Urinary tract infection | 0.38 | 0.05 |
| Pansinusitis/Sinusitis | -0.37 | 0.12 |
| Empyema/Lung abscess | 0.19 | 0.08 |
| Other infections | 0.55 | 0.10 |
| ICU variables |  |  |
| Intensive care unit (ICU) | 0.96 | 0.06 |
| Intensive care unit (observation, CVICU) | 0.34 | 0.06 |
| Markers of initial severity^a^ |  |  |
| Unable to take oral medications | 0.44 | 0.05 |
| Acetaminophen | -0.14 | 0.02 |
| Unfractionated heparin prophylaxis | 0.03 | 0.06 |
| Low molecular weight heparin prophylaxis | -0.05 | 0.04 |
| Dobutamine | 0.26 | 0.07 |
| Ketorolac | -0.40 | 0.07 |
| Foley catheter | 0.64 | 0.07 |
| Bicarbonate | 0.53 | 0.03 |
| Vasopressors | 0.54 | 0.03 |
| Benzodiazepenes | 0.33 | 0.07 |
| Arterial & Venous blood gas | 0.72 | 0.04 |
| Sputum culture | -0.16 | 0.03 |
| Urine culture | 0.04 | 0.02 |
| Pleural fluid analysis | 0.21 | 0.09 |
| Brain natriuretic peptide | 0.05 | 0.04 |
| Abdominal CT | -0.11 | 0.03 |
| Non-invasive ventilation | 0.44 | 0.03 |
| Invasive mechanical ventilation | 0.37 | 0.03 |
| Platelets | 1.07 | 0.25 |
| Arterial line | 0.41 | 0.12 |
| Vancomycin, linezolid, or quinupristin/dalfopristin | 0.32 | 0.03 |
| Anti-pseudomonal cephalosporin, carbapenem, beta-lactam, or aztreonam | 0.34 | 0.06 |
| Respiratory quinolone | 0.18 | 0.03 |
| Macrolide or respiratory quinolone | -0.26 | 0.03 |
| Markers of acute or chronic disease^a^ |  |  |
| Opiates | 0.74 | 0.06 |
| Zolpidem | -0.14 | 0.05 |
| Loop diuretics | 0.33 | 0.03 |
| Ipratropium | 0.07 | 0.02 |
| Non-steroidal anti-inflammatory drugs | -0.15 | 0.05 |
| Insulin | 0.19 | 0.04 |
| Interactions |  |  |
| Congestive heart failure*Hypothyroidism | 0.27 | 0.07 |
| Pulmonary circulation disease*Hypertension | 0.17 | 0.08 |
| Hypertension*Other neurological disorders | 0.30 | 0.06 |
| Hypertension*Chronic pulmonary disease | 0.14 | 0.04 |
| Hypertension*Diabetes | 0.16 | 0.05 |
| Hypertension*Metastatic cancer | 0.31 | 0.09 |
| Hypertension*Solid tumor without metastasis | 0.22 | 0.10 |
| Hypertension*Rheumatoid arthritis/Collagen vascular disease | 0.38 | 0.13 |
| Hypertension*Deficiency anemias | 0.23 | 0.05 |
| Other neurological disorders*CKD stage 5 | 1.54 | 0.46 |
| Diabetes*Weightloss | 0.22 | 0.07 |
| Diabetes*Drug abuse | 0.51 | 0.24 |
| Diabetes*Depression | 0.17 | 0.08 |
| Hypothyroidism*Depression | 0.35 | 0.09 |
| Lymphoma*Obesity | 0.95 | 0.31 |
| Obesity*Deficiency anemias | 0.38 | 0.09 |
| Deficiency anemias*CKD stage 3 | -0.30 | 0.11 |
| 18 - 24 years*Pulmonary circulation disease | 1.52 | 0.55 |
| 25 - 34 years*Pulmonary circulation disease | 0.63 | 0.43 |
| 35 - 44 years*Pulmonary circulation disease | -0.03 | 0.30 |
| 45 - 54 years*Pulmonary circulation disease | 0.45 | 0.17 |
| 55 - 64 years*Pulmonary circulation disease | 0.01 | 0.14 |
| 65 - 74 years*Pulmonary circulation disease | -0.04 | 0.11 |
| 75 - 84 years*Pulmonary circulation disease | -0.22 | 0.10 |
| 18 - 24 years*Hypertension | -1.47 | 1.04 |
| 25 - 34 years*Hypertension | -0.18 | 0.32 |
| 35 - 44 years*Hypertension | 0.05 | 0.17 |
| 45 - 54 years*Hypertension | -0.24 | 0.10 |
| 55 - 64 years*Hypertension | -0.31 | 0.07 |
| 65 - 74 years*Hypertension | -0.22 | 0.06 |
| 75 - 84 years*Hypertension | -0.06 | 0.05 |
| 18 - 24 years*Metastatic cancer | 2.97 | 1.03 |
| 25 - 34 years*Metastatic cancer | 2.05 | 0.48 |
| 35 - 44 years*Metastatic cancer | 1.89 | 0.28 |
| 45 - 54 years*Metastatic cancer | 1.27 | 0.19 |
| 55 - 64 years*Metastatic cancer | 0.84 | 0.16 |
| 65 - 74 years*Metastatic cancer | 0.63 | 0.15 |
| 75 - 84 years*Metastatic cancer | 0.45 | 0.15 |
| race_other*CKD stage 3 | 0.40 | 0.14 |
| race_black*CKD Stage 3 | -0.34 | 0.18 |
| race_hispanic*CKD stage 3 | 0.15 | 0.28 |
| Beta blocker*Anti-depressants | 0.20 | 0.06 |
| Digoxin*Carvedilol | -0.34 | 0.11 |
| Warfarin*Unfractionated heparin treatment | -0.53 | 0.22 |
| Ferrous sulphate*Packed red blood cells | -0.52 | 0.14 |
| Race_other*Anti-depressants | 0.16 | 0.06 |
| Race_black*Anti-depressants | -0.06 | 0.10 |
| Race_hispanic*Anti-depressants | 0.27 | 0.13 |
| Congestive heart failure*ICU | -0.21 | 0.05 |
| Urinary tract infection*ICU | -0.15 | 0.05 |
| Hypertension*Unable to take oral medications | 0.18 | 0.05 |
| Chronic pulmonary disease*Unable to take oral medications | -0.15 | 0.05 |
| ICU*Unable to take oral medications | -0.26 | 0.05 |
| 18 - 24 years*Low molecular weight heparin prophylaxis | 0.56 | 0.32 |
| 25 - 34 years*Low molecular weight heparin prophylaxis | -0.37 | 0.23 |
| 35 - 44 years*Low molecular weight heparin prophylaxis | -0.19 | 0.14 |
| 45 - 54 years*Low molecular weight heparin prophylaxis | -0.35 | 0.09 |
| 55 - 64 years*Low molecular weight heparin prophylaxis | -0.20 | 0.07 |
| 65 - 74 years*Low molecular weight heparin prophylaxis | -0.11 | 0.06 |
| 75 - 84 years*Low molecular weight heparin prophylaxis | -0.10 | 0.05 |
| 18 - 24 years*Benzodiazepenes | 0.33 | 0.35 |
| 25 - 34 years*Benzodiazepenes | -0.11 | 0.23 |
| 35 - 44 years*Benzodiazepenes | 0.10 | 0.14 |
| 45 - 54 years*Benzodiazepenes | -0.03 | 0.10 |
| 55 - 64 years*Benzodiazepenes | -0.13 | 0.08 |
| 65 - 74 years*Benzodiazepenes | -0.20 | 0.08 |
| 75 - 84 years*Benzodiazepenes | -0.19 | 0.07 |
| Hypertension*Benzodiazepenes | 0.13 | 0.05 |
| Unable to take oral medications*Benzodiazepenes | -0.19 | 0.05 |
| 18 - 24 years*Foley catheter | 0.37 | 0.37 |
| 25 - 34 years*Foley catheter | 0.10 | 0.26 |
| 35 - 44 years*Foley catheter | 0.50 | 0.15 |
| 45 - 54 years*Foley catheter | 0.17 | 0.10 |
| 55 - 64 years*Foley catheter | 0.02 | 0.08 |
| 65 - 74 years*Foley catheter | 0.02 | 0.07 |
| 75 - 84 years*Foley catheter | 0.01 | 0.06 |
| ICU*Foley catheter | -0.29 | 0.05 |
| Unable to take oral medications*Foley catheter | -0.15 | 0.05 |
| Congestive heart failure*Loop diuretics | -0.27 | 0.05 |
| Foley catheter*Loop diuretics | -0.23 | 0.05 |
| Diabetes*Insulin | -0.18 | 0.05 |
| ICU*Insulin | -0.13 | 0.04 |
| 18 - 24 years*Opiates | -0.67 | 0.34 |
| 25 - 34 years*Opiates | -0.47 | 0.21 |
| 35 - 44 years*Opiates | -0.18 | 0.14 |
| 45 - 54 years*Opiates | -0.28 | 0.09 |
| 55 - 64 years*Opiates | -0.22 | 0.07 |
| 65 - 74 years*Opiates | -0.15 | 0.07 |
| 75 - 84 years*Opiates | -0.20 | 0.06 |
| ICU*Opiates | -0.21 | 0.05 |
| Unable to take oral medications*Opiates | 0.33 | 0.05 |
| Benzodiazepenes*Opiates | -0.14 | 0.05 |
| 18 - 24 years*Brain natriuretic peptide | 0.30 | 0.37 |
| 25 - 34 years*Brain natriuretic peptide | 0.74 | 0.21 |
| 35 - 44 years*Brain natriuretic peptide | 0.08 | 0.14 |
| 45 - 54 years*Brain natriuretic peptide | 0.16 | 0.09 |
| 55 - 64 years*Brain natriuretic peptide | 0.09 | 0.07 |
| 65 - 74 years*Brain natriuretic peptide | 0.12 | 0.06 |
| 75 - 84 years*Brain natriuretic peptide | 0.05 | 0.05 |
| Insulin*Brain natriuretic peptide | -0.16 | 0.04 |
| Foley catheter*Urine culture | -0.15 | 0.05 |
| Congestive heart failure*Arterial & Venous blood gas | -0.16 | 0.05 |
| Chronic pulmonary disease*Arterial & Venous blood gas | -0.14 | 0.04 |
| Urinary tract infection*Arterial & Venous blood gas | -0.19 | 0.05 |
| ICU*Arterial & Venous blood gas | -0.29 | 0.05 |
| Opiates*Arterial & Venous blood gas | -0.10 | 0.05 |
| Hypertension*Vancomycin, linezolid, or quinupristin/dalfopristin | 0.18 | 0.05 |
| Paralysis*Vancomycin, linezolid, or quinupristin/dalfopristin | -0.33 | 0.11 |
| Other neurological disorders*Vancomycin, linezolid, or quinupristin/dalfopristin | -0.18 | 0.06 |
| Liver disease*Vancomycin, linezolid, or quinupristin/dalfopristin | 0.33 | 0.13 |
| Metastatic cancer*Vancomycin, linezolid, or quinupristin/dalfopristin | -0.21 | 0.09 |
| Weight loss*Vancomycin, linezolid, or quinupristin/dalfopristin | -0.19 | 0.07 |
| ICU(observation CVICU)*Vancomycin, linezolid, or quinupristin/dalfopristin | -0.25 | 0.07 |
| 18 - 24 years*Anti-pseudomonal cephalosporin, carbapenem, beta-lactam, or aztreonam | 0.19 | 0.33 |
| 25 - 34 years*Anti-pseudomonal cephalosporin, carbapenem, beta-lactam, or aztreonam | 0.62 | 0.22 |
| 35 - 44 years*Anti-pseudomonal cephalosporin, carbapenem, beta-lactam, or aztreonam | 0.48 | 0.14 |
| 45 - 54 years*Anti-pseudomonal cephalosporin, carbapenem, beta-lactam, or aztreonam | 0.30 | 0.09 |
| 55 - 64 years*Anti-pseudomonal cephalosporin, carbapenem, beta-lactam, or aztreonam | 0.30 | 0.07 |
| 65 - 74 years*Anti-pseudomonal cephalosporin, carbapenem, beta-lactam, or aztreonam | 0.14 | 0.06 |
| 75 - 84 years*Anti-pseudomonal cephalosporin, carbapenem, beta-lactam, or aztreonam | 0.13 | 0.05 |
| Female*Anti-pseudomonal cephalosporin, carbapenem, beta-lactam, or aztreonam | 0.13 | 0.04 |
| Hypertension*Anti-pseudomonal cephalosporin, carbapenem, beta-lactam, or aztreonam | 0.13 | 0.05 |
| Metastatic cancer*Anti-pseudomonal cephalosporin, carbapenem, beta-lactam, or aztreonam | -0.28 | 0.10 |
| Solid tumor without metastasis*Anti-pseudomonal cephalosporin, carbapenem, beta-lactam, or aztreonam | -0.32 | 0.10 |
| Weight loss*Anti-pseudomonal cephalosporin, carbapenem, beta-lactam, or aztreonam | -0.24 | 0.07 |
| CKD stage 3*Anti-pseudomonal cephalosporin, carbapenem, beta-lactam, or aztreonam | -0.30 | 0.10 |
| Urinary tract infection*Anti-pseudomonal cephalosporin, carbapenem, beta-lactam, or aztreonam | -0.22 | 0.05 |
| Other infection*Anti-pseudomonal cephalosporin, carbapenem, beta-lactam, or aztreonam | -0.31 | 0.12 |
| ICU*Anti-pseudomonal cephalosporin, carbapenem, beta-lactam, or aztreonam | -0.26 | 0.04 |
| Unfractionated heparin prophylaxis*Anti-pseudomonal cephalosporin, carbapenem, beta-lactam, or aztreonam | -0.12 | 0.06 |
| Foley catheter*Anti-pseudomonal cephalosporin, carbapenem, beta-lactam, or aztreonam | -0.23 | 0.05 |
| Arterial line*Anti-pseudomonal cephalosporin, carbapenem, beta-lactam, or aztreonam | -0.33 | 0.14 |
| Opiates*Anti-pseudomonal cephalosporin, carbapenem, beta-lactam, or aztreonam | -0.15 | 0.04 |
| Unfractionated heparin prophylaxis*Respiratory quinolone | -0.12 | 0.05 |
| Doxazosin*Macrolide or respiratory quinolone | 1.02 | 0.39 |

*p* ≤ .05

^a^within first 48 hours after admission
